# Action on waiting list for Speech Therapy in the Unified Health System (SUS) in a medium-sized Brazilian municipality

**DOI:** 10.1590/2317-1782/e20250038en

**Published:** 2026-05-08

**Authors:** Thamires Maria de Araújo Salles, Mayara Gabriela Pereira dos Santos, Rafaella de Góes Moraes, Matheus Francoy Alpes, Amanda Tragueta Ferreira-Vasques

**Affiliations:** 1 Universidade de Sorocaba – UNISO - Sorocaba (SP), Brasil.; 2 Instituto de Saúde de Nova Friburgo – ISNF, Universidade Federal Fluminense – UFF - Nova Friburgo (RJ), Brasil.

**Keywords:** Speech, Language and Hearing Sciences, Primary Attention, Unified Health System, Health Services, Waiting Lists, Public Health

## Abstract

**Purpose:**

To characterize the profile and demands of individuals on the waiting list for speech-language pathology care in a medium-sized Brazilian municipality.

**Methods:**

Descriptive study, carried out between July 2023 and July 2024, through telephone contact with individuals awaiting speech-language pathology care on the waiting list according to Center for Regulation and Evaluation and Control Division of a municipality in the interior of São Paulo. The list included individuals referred by the Primary Care network between February 2018 and December 2022. Information on sex, age, initial and current complaint, service area, and status in the queue (waiting or scheduled) were updated, collected by students under the guidance of professors of a Speech Therapy course in the aforementioned municipality. Data were analyzed using descriptive statistics.

**Results:**

Of the 393 individuals, 147 were excluded from the list (106 for no longer presenting demand and 41 for changing location). Among the remaining 246, ages ranged from 1 to 81 years, 71.9% were children up to 11 years old and 64.2% were male. In 26.8% of cases, there was a discrepancy between the current complaint and that on the referral form. The main demand was child language (83.9%). After organizing the information, 65.9% were scheduled for speech therapy.

**Conclusion:**

Updating the waiting list made it possible to identify the profile of individuals and their speech therapy demands, contributing to better planning and provision of services, in addition to helping to reduce the waiting list.

## INTRODUCTION

Speech-Language-Hearing (SLH) Pathology is the area responsible for the prevention, assessment, diagnosis, and treatment of human communication disorders, including language, speech, voice, hearing, and orofacial functions^(1)^. Its inclusion in different levels of healthcare of the Brazilian Unified Health System (SUS) is essential to guarantee comprehensive care, helping to improve users’ quality of life, social inclusion, and functioning^(2)^.

Healthcare is structured in primary, secondary, and tertiary levels, which are dynamically and complementarily interrelated. In Brazil, they are organized through the policies for basic or primary healthcare (PNAB)^(3)^, specialized healthcare (PNAES)^(4)^, and hospital care (PNHOSP)^(5)^. Since the real complexity of healthcare requires the articulation of diverse knowledge, practices, and technologies due to the diversity of user needs, this situation implies the need to combine soft technologies (such as bonding, support, and qualified listening) with soft-hard technologies (such as work processes and clinical protocols) and hard technologies (such as high-precision equipment, sophisticated examinations, and hospital interventions)^(6)^. Each level of care incorporates different degrees of complexity, requiring effective integration between services to ensure continuous and comprehensive care^(7)^.

Estimates that identify and characterize users and their needs are essential to strengthen the inclusion of SLH pathologists at these different levels of care and complexity. This information is crucial for systematizing criteria for organization and regulation of the various health sectors, contributing to the management of waiting lists^(8-10)^.

The long periods users spend on waiting lists without qualified care or guidance contribute to a poor prognosis of their condition, possibly worsening their status or complaints, constituting an important cause for individuals to give up on the care they need and remain with their health problems^(11)^.

Updating waiting lists allows for the analysis of inaccuracies in the planning and management of service distribution that may interfere with the waiting time for a particular type of care^(12)^. Thus, this study is justified by the need to produce information that improves the management of waiting lists and expands qualified access to SLH care in SUS. Thus, this study aimed to characterize the profile and needs of individuals on the waiting list for SLH care in a medium-sized Brazilian municipality.

## METHODS

This study was submitted to and approved by the Human Research Ethics Committee of the University of Sorocaba (UNISO), under number 6.431.411. All participants signed an informed consent form, following resolution 466/2012 on research ethics by the Brazilian National Research Ethics Commission – CONEP. The researchers (undergraduate SLH students) conducted this descriptive study in July 2023 and July 2024 for their senior writing project and outreach program, linked to a private university in a municipality in inland São Paulo. All research was supervised by the professors in charge.

Initially, the researchers held a meeting with the staff of the Regulation Center and Evaluation and Control Division (CREDAC), responsible for distributing vacancies in the municipality where the research was developed, to access the waiting list of individuals in need of public SLH services. These professionals were not SLH pathologists, and the waiting list was created from referrals from basic/primary healthcare between February 2018 and December 2022.

The selection was by convenience^(13)^ because the university knew about the existence of an SLH waiting list in the said municipality, as it inquired with the Municipal Health Department about the unmet need for SLH services. A Microsoft Excel spreadsheet was initially created with information on the identification and registration number of candidates for SLH services, as well as their sex, age, and initial complaint. Other details were also included to help resolve the candidate's situation, namely: the SLH subarea capable of addressing the complaint and the action to be taken by the professionals (continue waiting, withdraw/cancel the request, or schedule an appointment).

The inclusion criteria for study participants were presence on the waiting list, having been referred by primary/basic healthcare, having a complete registration (filling in all mandatory fields for information processing), and agreeing to participate by signing an informed consent form. The exclusion criteria were the absence or unavailability of telephone contact and candidates not residing in the municipality in question.

Data were collected through telephone contact made at CREDAC. The researchers had been previously trained to contact the users. They initially contacted users by telephone to invite them to participate in the research and later sent them an informed consent form for their agreement. Then, their data were updated in the Microsoft Excel spreadsheet. Only participants who agreed to the informed consent form were counted in this research.

The study participants were characterized and distributed into groups according to age range, based on specialized literature^(10)^ (C – childhood: birth to 11 years, A – adolescence: 11 to 20 years, YM - young/middle-aged adults: 20 to 65 years, OA – older adults: 65 years or more). In the case of minors, contact was made with their parents or guardians.

The data obtained and organized in a Microsoft Excel spreadsheet were analyzed using descriptive statistics with absolute (total) and relative (percentage) values. This approach allowed us to identify relevant patterns and characteristics of the sample without resorting to inferential analysis, since the focus of the study was to understand this population’s needs, without statistical generalizations to other populations.

## RESULTS

The researchers contacted 418 users. Altogether, 393 agreed to participate, but 147 were removed from the study – 106 due to a lack of current need (36 had already received care at another public or private facility, and 70 had their needs met naturally), and 41 due to a change of city, state, or country.

**Figure 1:**
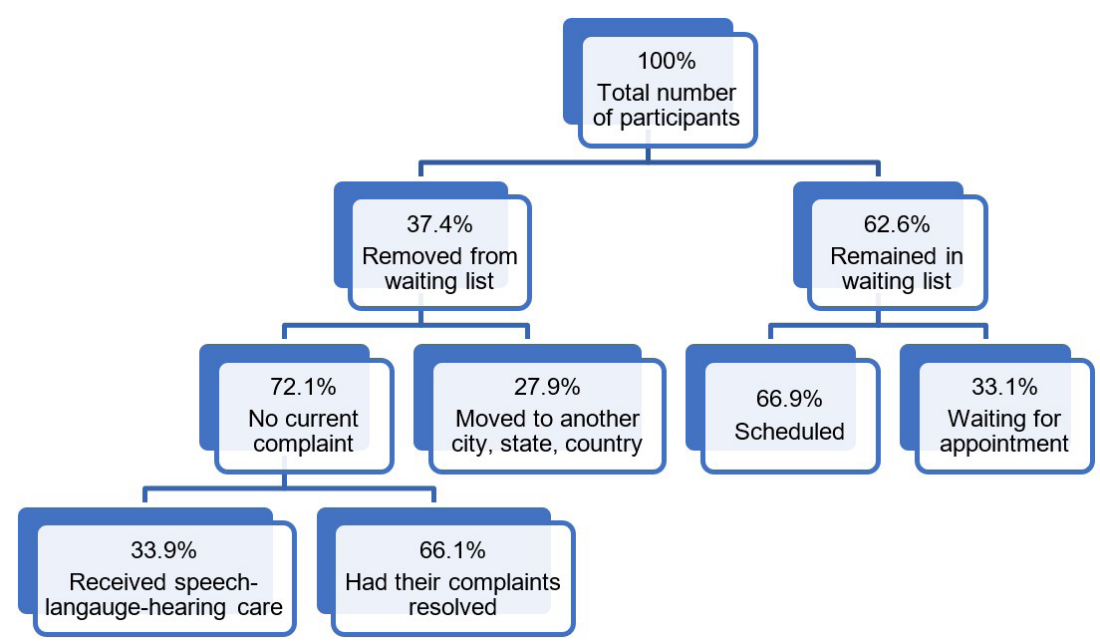
Flowchart of research data

The situation of 246 individuals was then addressed in detail. Each one had their current complaint analyzed to indicate the SLH subarea (specific area) that could meet their needs and define their status on the waiting list. After processing the information, 162 were scheduled for SLH care, and 84 continued to wait for scheduling (Figure 1).

Developed by the authors

Among the users referred for SLH care, 158 were male, and 88 were female, with a wide age range, from 1 year and 9 months to 81 years and 1 month. They were distributed as follows: 177 children, 35 adolescents, 20 young and middle-aged adults, and 14 older adults.

The complaint of 180 users remained unaltered from the original one, whereas 66 had a different complaint from what was stated in the referral form for SLH care. As for SLH subareas, 217 needed care in only one area, and 29 in two or three areas.

Among the 217 individuals awaiting care in only one subarea, the prevalent SLH complaint concerned child language (182 individuals), followed by ​​adult language (nine individuals), adult dysphagia (seven), voice (five), child dysphagia, audiology (four each), oral-motor therapy, and otoneurology (three each).

Among the 29 individuals awaiting SLH care in two or three areas, a higher prevalence was observed in audiology and child language (16 individuals), adult dysphagia and adult language (five), dysphagia and language (two), and child language and oral-motor therapy (two). One child had simultaneous complaints regarding oral-motor therapy, language, and audiology.

Table 1 presents all the data described above.

**Table 1:** Data obtained from the analysis of the waiting list for speech-language-hearing services

**Variables**	**%**
**Sex**	Males – 64.2%
	Females – 35.8%
**Age range**	Childhood – 71.9%
	Adolescence – 14.2%
	Young and middle-aged adults – 8%
	Older adults – 5.9%
**Initial complaint**	Unchanged initial complaint – 73.2%
	With changes from initial complaint – 26.8%
**Number of areas whose services were needed**	One SLH area – 88.2%
	Two or three SLH areas – 11.8%
**Individuals waiting for treatment in one SLH area**	Child language – 83.9%
	Adult language – 4.2%
	Adult dysphagia – 3.8%
	Voice – 2.1%
	Audiology – 1.6%
	Child dysphagia – 1.6%
	Oral-motor therapy – 1.4%
	Otoneurology - 1.4%
**Individuals waiting for treatment in two or three SLH areas**	Audiology and child language – 55.2%
	Adult dysphagia and language – 17.2%
	Child dysphagia and language – 7%
	Child language and oral-motor therapy – 7%
	Audiology and adult language – 3.4%
	Adult dysphagia and voice - 3.4%
	Audiology and adult dysphagia and language – 3.4%
	Audiology, oral-motor therapy, and child language – 3.4%

Developed by the authors

**Caption:** % - Percentage; SLH – speech-language-hearing

## DISCUSSION

Few studies approach factors related to SLH waiting lists. A Brazilian study addressed the waiting list of an SLH teaching clinic and proposed solutions such as referring individuals to other referral units and establishing more assertive criteria for care, facilitating the service’s internal organization^(11)^. Despite the practical relevance of waiting lists in public services, much of the existing scientific production comes from teaching clinics, environments more favorable to research because they are linked to universities. On the other hand, professionals who work directly in public services, although they experience the impacts of unmet demand daily, are overburdened and have little or no institutional structure to develop scientific research. This lack of production may also reflect the lack of appreciation for research in the daily routine of the SUS and the invisibility of the topic on the agenda of managers. The low systematization of data and the absence of specific indicators exacerbate this scenario, hindering accurate diagnoses and effective planning actions, making it urgent to recognize waiting lists not just as operational tools but as indicators of equity and access to services.

This research enabled the removal of individuals from the list at the first telephone contact due to the current absence of demand (complaint) or a change of location (city, state, or country). This clearly indicates that data may become outdated or changed, and the complaint of candidates for SLH care may change due to prolonged wait^(14,15)^.

There was a predominance of males, which can be justified by the greater occurrence of neurodevelopmental delays and changes in early childhood, adulthood, and old age in this population^(10,16,17)^. The variation in the age of the population awaiting SLH care can be justified by several factors, including biological aspects, such as genetic and social predispositions, which influence the development and perception of disorders^(18)^.

Another relevant factor is the breadth of SLH subareas, enabling interventions from birth to senescence^(19)^, especially in old age^(20)^. Thus, SLH practice is distributed throughout the life cycle. Oral and written language, oral-motor skills, and dysphagia stand out in childhood; voice and written language in adolescence; voice and language in adulthood (usually in post-stroke or traumatic brain injury rehabilitation); and the maintenance of cognitive and language skills and treatment of dysphagia in older adults^(18,20)^.

The analysis of the complaints of individuals on the waiting list revealed that most did not have any change, indicating stability in the needs of individuals throughout the waiting period. However, the complaints of some had worsened. The waiting list that was the subject of this study contained individuals who had been waiting for care since 2018 – i.e., approximately 5 years of waiting, which in a way agrees with other studies regarding waiting lists: maintaining a complaint can mean a poor prognosis^(11)^. From our point of view, the fact that it is a quarter of the list justifies the relevance of continuing studies on this topic.

Analysis of referrals from primary healthcare professionals revealed that the majority were directed to the subarea of ​​child language, reinforcing what has already been evidenced in other Brazilian studies: the high demand for care for this population^(17,21,22)^. There was also a predominance of referrals focused on isolated subareas, which reveals limitations in these professionals' understanding of SLH practice^(23)^. SLH pathologists are responsible for disseminating the scope of their area to other health and education professionals, among other sectors, particularly highlighting the importance of a comprehensive SLH approach.

Most individuals awaiting SLH services in two or three areas needed care in audiology and child language, followed by adults awaiting services in adult language and dysphagia, and children with dysphagia and language and oral-motor issues. These data reveal the importance of generalist training^(24)^ to ensure comprehensive SLH practice and encompassing, efficient care. This approach allows individuals' needs to be assessed and met holistically, considering the interrelationship between different aspects involved in communication and other oral functions addressed in SLH intervention.

SLH pathologists, as health professionals working in the public service, must carefully observe the specific needs and characteristics of the population they serve and/or seek their care. They must consider current health policies critically and thoughtfully, continuously seeking to understand how these guidelines can influence access, quality, and effectiveness of the services^(15)^. Furthermore, SLH pathologists are responsible for adapting their practices and interventions to better meet the needs of the population, considering socioeconomic, cultural, and educational aspects that may impact their communicative health.

After addressing the complaints of individuals on the waiting list, there was a significant reduction in the number of users in this condition. This demonstrates the positive impact of SLH intervention on the planning and management of demand for care in the area, which contributes to more timely and comprehensive access to SLH services. Involving SLH pathologists beyond clinical intervention, such as carrying out regulation or guiding and discussing with those who do so, brings resolution. It was possible to make more assertive appointments and update the individuals' data, which optimized the waiting list. In other words, this action qualified the treatment of the waiting list, categorized the individuals' needs better, and thus organized the municipality’s SLH service better.

It is worth noting that this study faced limitations, such as 1) having been carried out in a single municipality (which limits generalization); 2) registering data with incomplete and outdated information; 3) excluding people because only telephone contact was permitted (people without telephones or with deactivated numbers were left out); and 4) using only descriptive statistical analysis.

Given the above, one of the main strategies to solve the problem portrayed in this study would be to invest in the training of primary care professionals to carry out more detailed and complete referrals, thus establishing more systematic and horizontal relationships within health teams^(25)^. It would also be important to avoid creating long waiting lists, include SLH pathologists in community health centers, and implement stimulation groups – a valuable complementary strategy, as it can minimize the impacts of delays in diagnosis or intervention^(26)^.

Further studies are needed to deepen the knowledge about the needs of individuals awaiting public SLH care, with in-person support and/or discussions with health teams (cooperation), enhancing access to timely and quality SLH care for the population that needs it.

## CONCLUSION

This study aimed to characterize individuals and their SLH needs and update the waiting list in the public sector of a medium-sized municipality, created over 5 years. The prevalence among the 246 individuals on the waiting list was early childhood (up to 11 years old) and male, with complaints in the subarea of language.

Hence, 162 appointments were scheduled, improving the service to reduce the waiting time for SLH care in the municipality. This type of research and action is configured as a provider of health data processing, which is an issue to be resolved by the municipal government and faced by health professionals, especially SLH pathologists.
