# Supplementary material for: Action on waiting list for Speech Therapy in the Unified Health System (SUS) in a medium-sized Brazilian municipality
Source: Codas. 2026 May 8;38(3):e20250038. doi: 10.1590/2317-1782/e20250038en (PMC13193191; doi:10.1590/2317-1782/e20250038en)
Supplement: Translation

# Translation: Ação sobre lista de espera para a Fonoaudiologia no Sistema Único de Saúde (SUS) em um município brasileiro de médio porte

**DOI:** 10.1590/2317-1782/e20250038pt

**Authors:** Thamires Maria de Araújo Salles, Mayara Gabriela Pereira dos Santos, Rafaella de Góes Moraes, Matheus Francoy Alpes, Amanda Tragueta Ferreira-Vasques

**Affiliations:** 1 Universidade de Sorocaba – UNISO - Sorocaba (SP), Brasil.; 2 Instituto de Saúde de Nova Friburgo – ISNF, Universidade Federal Fluminense – UFF - Nova Friburgo (RJ), Brasil.

**Keywords:** Fonoaudiologia, Atenção Primária, Sistema Único de Saúde, Serviços de Saúde, Listas de Esperas, Saúde Pública

## Abstract

**Objetivo:**

Caracterizar o perfil e as demandas dos indivíduos na lista de espera por atendimento fonoaudiológico em um município brasileiro de médio porte.

**Métodos:**

Estudo descritivo, realizado entre julho de 2023 e julho de 2024, por meio de contato telefônico com indivíduos que aguardavam atendimento fonoaudiológico na lista de de espera de acordo com o Centro de Regulação e Divisão de Avaliação e Controle de um município do interior paulista. A lista incluía pessoas encaminhadas pela rede de Atenção Básica entre fevereiro de 2018 e dezembro de 2022. Foram atualizadas informações sobre sexo, idade, queixa inicial e atual, área de atendimento e situação na fila (aguardando ou agendado), coletadas por estudantes sob orientação de docentes de um curso de Fonoaudiologia do referido município. Os dados foram analisados por estatística descritiva.

**Resultados:**

Dos 393 indivíduos, 147 foram excluídos da lista (106 por não apresentarem mais demanda e 41 por mudança de local). Entre os 246 restantes, as idades variaram entre 1 e 81 anos, sendo 71,9% crianças até 11 anos e 64,2% do sexo masculino. Em 26,8% dos casos, houve divergência entre a queixa atual e a da guia de encaminhamento. A principal demanda foi linguagem infantil (83,9%). Após organização das informações, 65,9% foram agendados para atendimento fonoaudiológico.

**Conclusão:**

A atualização da lista de espera possibilitou identificar o perfil dos indivíduos e suas demandas fonoaudiológicas, contribuindo para o melhor planejamento e oferta de serviços, além de colaborar para a redução da fila de espera.

## INTRODUÇÃO

A Fonoaudiologia é a área responsável pela prevenção, avaliação, diagnóstico e tratamento dos distúrbios da comunicação humana, incluindo linguagem, fala, voz, audição e funções orofaciais^(1)^. No Sistema Único de Saúde (SUS), a sua inserção nos diferentes níveis de atenção à saúde é essencial para garantir o cuidado integral, contribuindo para a melhoria da qualidade de vida, inclusão social e funcionalidade dos usuários^(2)^.

A Atenção à Saúde é estruturada em níveis — primário, secundário e terciário — que se inter-relacionam de forma dinâmica e complementar; no Brasil, tal organização dá-se por meio das Políticas de Atenção Básica ou Primária à Saúde (PNAB)^(3)^, Atenção Especializada em Saúde (PNAES)^(4)^ e Atenção Hospitalar (PNHOSP)^(5)^. Visto que a complexidade real do cuidado em saúde, exige a articulação de diversos saberes, práticas e tecnologias frente a diversidade de demandas dos usuários, tal situação implica a necessidade de combinar tecnologias leves, como o vínculo, o acolhimento e a escuta qualificada, com tecnologias leves-duras, como os processos de trabalho e protocolos clínicos, e ainda com tecnologias duras, como equipamentos de alta precisão, exames sofisticados e intervenções hospitalares^(6)^. Cada nível de atenção incorpora diferentes graus de complexidade, exigindo integração efetiva entre os serviços para garantir a continuidade e a integralidade do cuidado^(7)^.

Para fortalecer a inclusão do fonoaudiólogo nestes diferentes níveis de atenção e complexidade, é essencial contar com estimativas que identifiquem e caracterizem os usuários e as suas demandas, sendo estas informações fundamentais para a sistematização de critérios de organização e regulação dos diversos setores da saúde, contribuindo para o manejo das listas de espera^(8-10)^.

Os longos períodos dos usuários nas listas de espera sem atendimento ou orientação qualificada contribuem para o mau prognóstico das demandas encontradas nos usuários e possível agravo dos quadros ou das queixas, configurando-se como causa importante para que os indivíduos desistam do atendimento pelo qual necessitam e permaneçam com os problemas de saúde^(11)^.

A atualização das listas de espera permite a análise das imprecisões encontradas no planejamento e na gestão da distribuição dos serviços que podem interferir no tempo de espera por determinado tipo de atendimento^(12)^. Assim, este estudo se justifica pela necessidade de produzir informações que contribuam para o aprimoramento da gestão das listas de espera e para a ampliação do acesso qualificado à Fonoaudiologia no SUS. Com base neste contexto, teve como objetivo caracterizar o perfil e as demandas dos indivíduos na lista de espera por atendimento fonoaudiológico de um município brasileiro de médio porte.

## MÉTODO

O presente estudo foi submetido e aprovado pelo Comitê de Ética em Pesquisa com Seres Humanos (CEP) da Universidade de Sorocaba (UNISO), sob número 6.431.411. Todos os participantes assinaram o Termo de Consentimento Livre e Esclarecido (TCLE), seguindo a resolução 466/2012, que se refere à ética em pesquisas da Comissão Nacional de Ética em Pesquisa – CONEP. O estudo é do tipo descritivo e foi realizado durante os meses de julho de 2023 e julho de 2024 por pesquisadores (estudantes de um Curso de Fonoaudiologia) para a finalidade de um Trabalho de Conclusão de Curso (TCC) e Projeto de Extensão Universitária, vinculados a uma Universidade privada de um município do interior do Estado de São Paulo. Toda a pesquisa foi supervisionada por docentes responsáveis.

Inicialmente, os pesquisadores realizaram uma reunião com os funcionários do Centro de Regulação e Divisão de Avaliação e Controle (CREDAC), responsáveis pela distribuição de vagas do município no qual a pesquisa foi desenvolvida, para acesso a lista de espera dos indivíduos que aguardavam por atendimento fonoaudiológico no setor público. Vale ressaltar que os referidos profissionais não eram profissionais fonoaudiólogos e que a lista de espera foi criada a partir de encaminhamentos advindos da Atenção Básica/Atenção Primária à Saúde, entre fevereiro de 2018 e dezembro de 2022.

A seleção foi por conveniência^(13)^, pois se sabia da existência da lista de espera para atendimento fonoaudiológico no referido município, uma vez que a Instituição de Ensino Superior sondou a Secretaria Municipal de Saúde sobre demanda reprimida para atendimento fonoaudiológico. Inicialmente, foi elaborada uma planilha do *Microsoft Excel*, incluindo informações relacionadas à identificação e número/cadastro do candidato a atendimento fonoaudiológico, assim como seu sexo, idade, queixa inicial. Além dessas informações, foram incluídas outras visando a resolutividade da situação do candidato a saber: queixa subárea da Fonoaudiologia capaz de responder à queixa e atitude a ser tomada pelos profissionais (continuar aguardando, retirada/cancelamento da solicitação ou agendamento para atendimento).

Os critérios de inclusão dos participantes deste estudo contemplaram: estar na lista de espera; ter sido encaminhado pela Atenção Básica/Primária e apresentar cadastro completo (preencher todos os campos obrigatórios para o tratamento das informações), assim como concordar com a participação por meio da assinatura do TCLE. Quanto aos critérios de exclusão, foram considerados: a ausência ou indisponibilidade de contato via telefone e o fato de o candidato ao atendimento não residir no município referido.

A coleta destas informações foi desenvolvida por meio de contato telefônico realizado no CREDAC e os pesquisadores tiveram um treinamento prévio para a realização do contato com os usuários. No contato telefônico, inicialmente era realizado o convite para participação da pesquisa e enviado o TCLE para concordância do indivíduo, e, após, realizada a atualização dos dados dos indivíduos na planilha do *Microsoft Excel*. Apenas os participantes que tiveram o aceite do TCLE foram contabilizados nesta pesquisa.

Os participantes do estudo foram caracterizados e distribuídos em grupos de acordo com a faixa etária, embasados em literatura especializada^(10)^ (I - Infância: nascimento aos 11 anos, A - Adolescência: 11 aos 20 anos, JM - Jovem adulto / meia idade: 20 aos 65 anos, PI – Pessoas idosas: 65 anos ou mais). Em caso de indivíduos menores de idade, o contato era realizado com o responsável legal.

Os dados obtidos e organizados em planilha do *Microsoft Excel* foram analisados por meio de estatística descritiva com valores absolutos (total) e relativos (porcentagem). Essa abordagem permitiu identificar padrões e características relevantes da amostra sem recorrer à análise inferencial, uma vez que o foco do estudo era compreender as demandas desta população, sem generalizações estatísticas para outras populações.

## RESULTADOS

Foram contatados 418 usuários, dos quais 393 aceitaram participar. Destes 393 indivíduos, 147 foram retirados do estudo, sendo 106 por ausência de demanda atual (36 já havia realizado o acompanhamento em outro local de atendimento público ou privado e 70 sanado as demandas naturalmente) e 41 por mudança de cidade, estado ou país.

Foi então, tratada detalhadamente a situação de 246 indivíduos. Cada um teve analisada sua queixa atual e indicada a subárea (área específica) da Fonoaudiologia, capaz de responder às suas necessidades, bem como situação na lista de espera. Após o tratamento das informações, 162 foram agendados para o atendimento fonoaudiológico e 84 continuaram aguardando por agendamento (Figura 1).

**Figura 1:** Fluxograma dos dados da pesquisa

Elaborado pelos autores

Dentre os usuários referenciados para a Fonoaudiologia, 158 eram do sexo masculino e 88 do sexo feminino, com ampla faixa etária distribuída entre 1 ano e 9 meses e 81 anos e 1 mês. A faixa etária dos indivíduos revelou a seguinte distribuição: 177 crianças, 35 adolescentes, 20 jovens adultos e adultos de meia-idade e 14 idosos.

Em relação à queixa, observou-se que 180 não tiveram alteração da original e 66 apresentaram queixa divergente da que constava na guia de encaminhamento para atendimento fonoaudiológico. Quanto às subáreas da Fonoaudiologia, 217 necessitavam de atendimento em apenas uma e 29 em duas ou três áreas.

Dentre os 217 indivíduos que aguardavam atendimento em apenas uma subárea, verificou-se que a queixa fonoaudiológica prevalente foi na de linguagem infantil (182 indivíduos). Em seguida, observou-se que as queixas eram na subárea de linguagem adulto (nove indivíduos), disfagia adulto (sete), voz (cinco), disfagia infantil e audiologia (quatro cada uma) e três indivíduos com queixa em motricidade orofacial e três em otoneurologia.

Entre os 29 indivíduos que aguardam atendimento fonoaudiológico em duas ou três áreas, observou-se maior prevalência nas áreas de audiologia e linguagem infantil (16 indivíduos), disfagia adulto e linguagem adulto (cinco), disfagia e linguagem (dois), linguagem infantil e motricidade orofacial (também dois indivíduos). Também foi observado a existência de uma criança com queixa simultânea de motricidade orofacial, linguagem e audiologia.

A Tabela 1 apresenta todos os dados descritos acima.

**Tabela 1:** Dados obtidos com a análise da lista de espera para atendimento fonoaudiológico

**Variáveis**	**%**
**Sexo**	Masculino – 64,2%
	Feminino - 35,8%
**Faixa etária**	Infância – 71,9%
	Adolescência - 14,2%
	Jovens adultos e pessoas de meia-idade- 8%
	Pessoas idosas – 5,9%
**Queixa inicial**	Sem alteração da queixa inicial – 73,2%
	Com alteração da queixa inicial – 26,8%
**Quantitativo de áreas que aguardavam atendimento**	Uma área da Fonoaudiologia – 88,2%
	Duas ou três áreas da Fonoaudiologia – 11,8%
**Indivíduos que aguardavam atendimento em uma área da Fonoaudiologia**	Linguagem infantil – 83,9%
	Linguagem adulto – 4,2%
	Disfagia adulto – 3,8%
	Voz – 2,1%
	Audiologia – 1,6%
	Disfagia infantil – 1,6%
	Motricidade orofacial – 1,4%
	Otoneurologia - 1,4%
**Indivíduos que aguardavam atendimento em duas ou três áreas da Fonoaudiologia**	Audiologia e Linguagem infantil – 55,2%
	Disfagia adulto e Linguagem adulto – 17,2%
	Disfagia infantil e Linguagem infantil – 7%
	Linguagem infantil e Motricidade orofacial – 7%
	Audiologia e Linguagem Adulto – 3,4%
	Disfagia Adulto e Voz - 3,4%
	Audiologia, Disfagia Adulto e Linguagem Adulto - 3,4%
	Audiologia, Motricidade orofacial e Linguagem infantil - 3,4%

Elaborado pelos autores

**Legenda:** % - Porcentagem

## DISCUSSÃO

São escassos os estudos que apontam fatores relacionados às listas de espera no âmbito da Fonoaudiologia. Um estudo brasileiro realizou o tratamento da lista de espera de uma Clínica-Escola de Fonoaudiologia e propôs como soluções, o encaminhamento dos indivíduos a outras unidades de referência e o estabelecimento de critérios mais assertivos para o atendimento, facilitando a organização interna do Serviço^(11)^. Apesar da relevância prática das listas de espera em serviços públicos, grande parte da produção científica existente é oriunda de Clínicas-Escola, ambientes mais favoráveis à pesquisa por estarem vinculados à Instituições de Ensino Superior. Por outro lado, os profissionais que atuam diretamente nos serviços públicos, embora vivenciem diariamente os impactos da demanda reprimida, encontram-se sobrecarregados e com pouca ou nenhuma estrutura institucional para desenvolver investigação científica. Essa ausência de produção pode refletir também a falta de valorização da pesquisa no cotidiano do SUS e a invisibilidade do tema na agenda dos gestores. A baixa sistematização de dados e a ausência de indicadores específicos agravam esse cenário, dificultando diagnósticos precisos e ações efetivas de planejamento, sendo urgente reconhecer a lista de espera como um indicador de acesso e equidade aos serviços e não apenas como uma ferramenta operacional.

Com o desenvolvimento desta pesquisa, ao primeiro contato telefônico, foram retirados indivíduos da lista devido à ausência atual de demanda (queixa) ou por mudança de local (cidade, estado ou país). Diante deste cenário é notório que, quando se há um tempo de espera prolongado, além dos dados ficarem desatualizados e ocorrerem mudanças quanto a essa questão, a queixa dos candidatos a atendimento fonoaudiológico também pode se alterar^(14,15)^.

Houve um predomínio de indivíduos do sexo masculino, o que pode ser justificado devido a maior ocorrência de atrasos e/ou alterações do neurodesenvolvimento na primeira infância, na fase adulta e no envelhecimento com esta população^(10,16,17)^. A variação na idade da população que aguarda atendimento fonoaudiológico pode ser justificada por diversos fatores, incluindo aspectos biológicos, como predisposições genéticas e sociais, que influenciam o desenvolvimento e a percepção dos transtornos^(18)^.

Outro fator relevante é a amplitude das subáreas englobadas na ciência fonoaudiológica, possibilitando intervenções desde o nascimento até a senescência^(19)^ e, sobretudo, na senilidade^(20)^. Portanto, a atuação fonoaudiológica se distribui ao longo do ciclo vital. Na infância, destacam-se a linguagem oral e escrita, motricidade orofacial e a disfagia; na adolescência, a voz e a linguagem escrita; na fase adulta, a voz e linguagem (geralmente na reabilitação pós-AVC ou traumatismos cranioencefálicos); e na senescência, incentivando a manutenção das habilidades cognitivas e de linguagem, bem como da disfagia^(18,20)^.

Quanto à análise das queixas dos indivíduos em lista de espera, observou-se que a maioria não apresentou modificação, indicando estabilidade das demandas dos indivíduos ao longo do período de espera, contudo, alguns tiveram agravamento de suas queixas. A lista de espera que foi objeto deste estudo continha indivíduos que aguardavam atendimento desde o ano de 2018, ou seja, aproximadamente cinco anos de espera, o que de certa forma concorda com outros estudos a respeito de listas de espera: manter queixa pode significar mau prognóstico^(11)^. Do nosso ponto de vista, o fato de ser um quarto da lista justifica a relevância em continuar os estudos sobre esta temática.

A análise dos encaminhamentos realizados por profissionais médicos da Atenção Básica/Primária revelou que a maioria foi direcionada à subárea de linguagem infantil, reforçando o que já foi evidenciado em outros estudos brasileiros -a alta demanda por atendimento a esta população^(17,21,22)^. Também houve predominância de encaminhamentos voltados para subáreas isoladas, o que revela limitações de entendimento desses profissionais quanto a atuação fonoaudiológica^(23)^. Cabe aos fonoaudiólogos divulgarem, junto aos demais profissionais da saúde, da educação e/ou outros setores, a abrangência da área e, sobretudo, destacar a importância da abordagem integral no campo fonoaudiológico.

Entre os indivíduos que aguardavam atendimento fonoaudiológico em duas ou três áreas, observa-se que a maioria aguardava atendimento em audiologia e linguagem infantil, seguidos dos adultos que aguardavam atendimento em linguagem adulto e disfagia, bem como o de crianças com disfagia e problemas de linguagem e motricidade orofacial. Tais dados revelam a importância de uma formação generalista^(24)^ para garantir uma atuação fonoaudiológica integral e um cuidado abrangente e eficiente. O enfoque permite que as necessidades dos indivíduos sejam avaliadas e atendidas de maneira holística, considerando as interrelações entre diferentes aspectos envolvidos na comunicação e nas demais funções orais (objetos da intervenção fonoaudiológica).

O fonoaudiólogo, como profissional de saúde inserido no serviço público, deve observar atentamente as necessidades e características específicas da população que atende e/ou que procura por atendimento. É essencial que ele adote uma postura crítica e reflexiva em relação às políticas de saúde vigentes, buscando continuamente compreender como essas diretrizes podem influenciar o acesso, a qualidade e a eficácia dos serviços oferecidos^(15)^. Além disso, cabe ao fonoaudiólogo adaptar suas práticas e intervenções para melhor atender a realidade da população, considerando aspectos socioeconômicos, culturais e educacionais que possam impactar a saúde comunicativa dos indivíduos.

Após o tratamento das queixas dos indivíduos em lista de espera, houve uma redução importante do número de usuários nessa condição. Esse dado demonstra o impacto positivo da intervenção fonoaudiológica no planejamento e na gestão da demanda por atendimento na área, o que contribui para um acesso mais oportuno e integral aos serviços fonoaudiológicos. Implicar o fonoaudiólogo para além da intervenção clínica, como por exemplo, executar a regulação ou orientar e discutir com quem a faz traz resolutividade. Foi possível realizar agendamentos mais assertivos, bem como a atualização dos dados dos indivíduos que produziu otimização da fila de espera. Em outras palavras, esta ação permitiu qualificar o tratamento da lista de espera, produziu melhor categorização das necessidades dos indivíduos e, consequentemente, melhor organização do serviço fonoaudiológico no município.

Vale destacar que este estudo enfrentou limitações como: 1) ter sido realizado em um único município (o que limita a generalização); 2) dados cadastrais com informações incompletas e desatualizadas; 3) exclusão de pessoas pelo fato de o contato ter sido apenas telefônico (ficaram de fora pessoas sem telefone ou com números desativados) e, por fim, 4) a análise estatística ter sido apenas descritiva.

Diante do exposto, uma das principais estratégias para solucionar a problemática retratada neste estudo, seria investir na capacitação de profissionais da Atenção Básica/Primária para a realização de encaminhamentos mais detalhados e completos, estabelecendo-se, assim, relações mais sistemáticas e horizontais dentro das equipes de saúde^(25)^. Além disso, para evitar a criação de listas de esperas volumosas, seria importante a presença de fonoaudiólogos nas unidades básicas de saúde (UBS) e a implementação de grupos de estimulação – uma estratégia complementar de grande valor à medida que pode minimizar os impactos de atrasos no diagnóstico ou na intervenção^(26)^.

É necessária a continuidade do estudo para aprofundar o conhecimento acerca das necessidades dos indivíduos que aguardam atendimento fonoaudiológico no setor público, com a realização de acolhimento presencial e/ou discussões com equipes de saúde (matriciamento), potencializando o acesso ao atendimento fonoaudiológico oportuno e de qualidade à população que dele necessita.

## CONCLUSÃO

Este estudo objetivou caracterizar os indivíduos e suas demandas fonoaudiológicas ao mesmo tempo que atualizou a lista de espera no setor público de um município de médio porte criada ao longo de cinco anos. Dos 246 indivíduos em lista de espera, a prevalência foi da primeira infância (até 11 anos) e do sexo masculino, com queixas na subárea de linguagem.

Foram agendados 162; aprimorou-se a oferta de serviços visando a redução do tempo de espera para atendimento fonoaudiológico no município. Este tipo de pesquisa e ação se configura como provedora do tratamento de dados em saúde, que não é apenas uma questão a ser resolvida pelo órgão administrativo da gestão municipal, mas também uma questão a ser enfrentada pelos profissionais de saúde, destacadamente o fonoaudiólogo.
